# Hypericin inhibits pathological retinal neovascularization in a mouse model of oxygen-induced retinopathy

**Published:** 2008-02-04

**Authors:** Akiko Higuchi, Haruhiko Yamada, Eri Yamada, Nobuo Jo, Miyo Matsumura

**Affiliations:** Department of Ophthalmology, Kansai Medical University, Osaka, Japan

## Abstract

**Purpose:**

Ocular neovascularization is a leading cause of blindness in ischemic retinopathies. Hypericin is an active ingredient in the medical herb St. John’s Wort (SJW). Because hypericin inhibits intracellular signaling pathways that are believed to participate in the regulation of angiogenesis, we investigated the actions of hypericin and SJW in retinal neovascularization, using a mouse model of oxygen-induced retinopathy (OIR).

**Methods:**

C57BL/6 neonatal mice were exposed to a 75% concentration of oxygen from postnatal day 7 (P7) to P12 and returned to room air from P12 to P17 to induce retinal neovascularization. SJW (15 mg/kg/day), hypericin (15, 45, or 135 μg/kg/day), or vehicle was given by gavage once a day for five days from P12 to P17. To quantify the area of retinal neovascularization and vasoobliteration, we stained retinas with isolectin B4 at P17. Phosphorylation of extracellular signal-regulated kinase (ERK) in ischemic retinas was determined by western blot analysis. To estimate retinal vascularization, we stained retinas with isolectin B4 at P7 after treatment with SJW, hypericin, or vehicle from P3 to P7.

**Results:**

Gavage administration of hypericin or SJW significantly inhibited the degree of retinal neovascularization, but did not affect the area of retinal vasoobliteration in a mouse model of OIR. Both SJW and hypericin had no effect on normal vascularization over the treatment time course. Treatment with SJW or hypericin reduced phosphorylation of ERK in the retina.

**Conclusions:**

These data suggest that hypericin and SJW reduce pathological retinal neovascularization and that administration of these agents could have clinical utility for treatment of ischemic retinopathies.

## Introduction

New blood vessel formation is an essential feature in the development of several pathological conditions including tumor growth, proliferative retinopathies, psoriasis, rheumatoid arthritis, osteoarthritis, and atherosclerosis [1]. Ocular neovascularization causes vision loss in various ischemic retinal disorders including retinopathy of prematurity and diabetic retinopathy [2,3]. Several angiogenic growth factors and cytokines that stimulate angiogenesis participate in the pathogenesis of retinal neovascularization [4-6]. Among them, vascular endothelial growth factor (VEGF), a potent endothelial cell-specific mitogen, plays a key role in regulation of new blood vessel growth in retina during pathological conditions [2,5].

VEGF binds to its receptors and stimulates a variety of signaling molecules, resulting in promotion of neovascularization [7-10]. Both extracellular signal-regulated kinase (ERK) and protein kinase C (PKC) are activated by VEGF and contribute to the induction of endothelial cell proliferation and migration, which are essential for regulation of angiogenesis [9,11-13]. Inhibition of PKC by tranilast, an antiallergic compound is associated with reduction of angiogenic activities in retinal microcapillary endothelial cells [12]. Intravitreous injection of ERK inhibitors leads to suppression of retinal neovascularization in a rat model of retinopathy of prematurity [14]. Therefore, therapies aimed at inhibiting VEGF-mediated signaling pathways may have efficacy in the treatment of pathological retinal neovascularization [6,15].

Hypericum perforatum, also known as St. John’s Wort (SJW), is a popular herbal supplement that is believed to exert antidepressant effects [16,17]. SJW also displays various pharmacological activities including antiviral and antineoplastic properties [16,17]. SJW contains various active constituents such as hypericin, hyperforin, tannins, and flavonoids [16,17]. Hypericin is a photodynamic compound that is one of the major active components of SJW. Hypericin has been shown to suppress several phosphorylation signaling pathways, including ERK and PKC in cultured cells [18,19]. These reports led us to hypothesize that SJW and its active component hypericin could negatively regulate ischemia-induced angiogenesis. However, the impact of hypericin or SJW on retinal neovascularization in vivo has not been examined previously. Thus, we investigated whether SJW or hypericin could modulate retinal neovascularization in a mouse model of ischemic retinopathy.

## Methods

### A mouse model of oxygen-induced retinopathy

All animal experiments were performed in accordance with the Association for Research in Vision and Ophthalmology Statement for the Use of Animals in Ophthalmic and Vision Research. The Committee of Animal Use of the Kansai Medical University approved the experimental protocol. (Adult male mice; n=15, adult female mice; n=30) C57BL/6 mice were obtained from CLEA Japan (Tokyo, Japan). Neonatal mice were subjected to oxygen-induced

retinopathy as described previously [20]. Briefly postnatal day 7 (P7) mice with their nursing mothers were exposed to 75%±2% oxygen (hyperoxia) for five days (P7–12) to produce retinal vasoobliteration, and returned to room air (normoxia) for five days (P12–17) to induce ischemic retinal neovascularization.

At P12, after pups were exposed to 75% oxygen, they were categorized into three groups: SJW (15 mg/kg/day, which is the approximately same dose used as a supplement for humans), hypericin (15, 45, and 135 µg/kg/day), and vehicle group. Hypericin purified from SJW was purchased from Alexis Biochemicals (Lausen, Switzerland). SJW formulations are standardized to include 0.3% concentration of total hypericin containing hypericin and pseudohypericin, and the amount of pseudohypericin in SJW is around two to four times higher than that of hypericin [17,21]. Thus, the concentration of hypericin found in SJW appears to range between 0.06 and 0.1%. The amount of hypericin (15 µg/kg/day) approximately corresponds to that present in the dose of SJW (15 mg/kg/day). SJW and hypericin were dissolved in dimethyl sulfoxide (DMSO) and diluted with water to adjust the final concentration of DMSO to 0.5%. Vehicle contained 0.5% concentration of DMSO. SJW, hypericin, or vehicle was placed in the stomach by gavage once a day for five days. At P17 after five days of gavage treatment, mice were sacrificed by intraperitoneal injection with an overdose of ketamine (300 mg/kg) and xylazine (30 mg/kg).

### Quantification of retinal vasoobliteration and neovascularization

Eyes were removed from mice at P17 and fixed in 4% paraformaldehyde for 1 h at 4 °C. The retinas were dissected. They were then stained with Griffonia Simplicifolia Isolectin B4 conjugated with Alexa Fluor 594 (Molecular Probes, Carlsbad, CA) for 16 h at 4 °C to detect vascular endothelial cells [22]. The retinas were washed with phosphate-buffered saline (PBS) for 2 h. Four radical cuts were made from the edge of each retina to the center, dividing the retina into four quadrants, before it was mounted in mounting media (Vector Laboratories, Burlingame, CA). Pictures of whole-mounts of retinas were taken at 5X magnification by fluorescence microscopy. Retinal segments were merged to generate a whole retinal image using Adobe^®^ Photoshop^®^ Version 7.0 (San Jose, CA). Vasoobliteration and neovascular tuft formation were quantified by comparing the number of pixels in the affected areas with the total number of pixels in the retina. Investigators were blinded to the mouse treatment.

**Figure 1:**
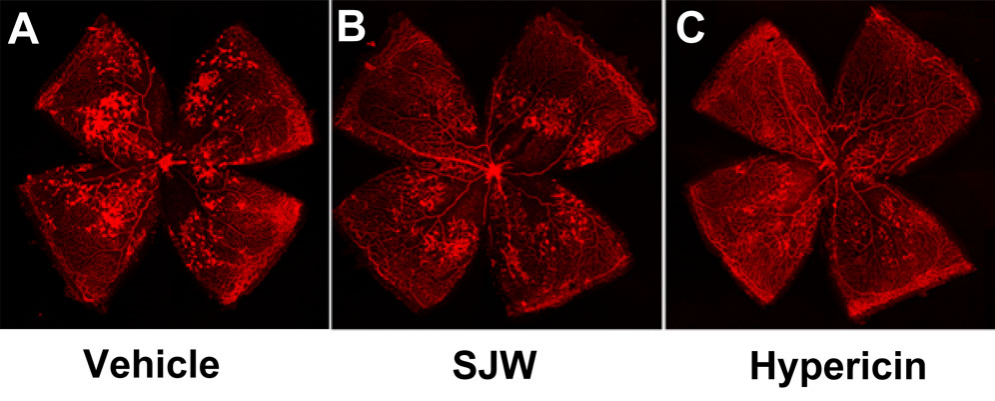
St. John’s Wort and hypericin inhibit ischemia-induced retinal vessel formations. Shown are representative photographs of retinas stained with isolectin B4. Mice at P7 were reared in 75% oxygen for five days followed by room air for five days. St. John’s Wort (**B**) (SJW; 15 mg/kg/day), hypericin (**C**) (45 µg/kg/day), and vehicle (**A**) were administered to mice by gavage from P12 to p17. Retinas were isolated and stained with isolectin B4 to detect vasoobliteration and neovascularization. Magnification is X 5.

### Quantification of retinal vascular development

SJW, hypericin, or vehicle was administered to mice by gavage once a day from P3 to P7. At P7, eyes were enucleated and fixed with 4% paraformaldehyde for 1 h at 4 °C. The retinas were washed with PBS then dissected and incubated for 16 h at 4 °C with biotinylated *Griffonia Simplicifolia* isolectin B4 (Vector Laboratories, Burlingame, CA) diluted in PBS containing 1% Triton X-100 (Sigma-Aldrich, St. Louis, MO). Retinas were washed with PBS followed by incubation with fluorescein-isothiocyanate-streptavidin (Dako Corp., Glostrup, Denmark) for 16 h at 4 °C. Radial cuts were made in the peripheral retina to allow whole mounting in mounting medium (Vector Laboratories). The whole-mounted retinas were analyzed by fluorescence microscopy. Vasculature length was measured from the optic disc to the edge of the vascular front in all four retinal quadrants using Image-Pro Plus software (Media Cybernetics, Silver Springs, MD) [6], and the average value was taken for each retina.

### Western blot analysis

Retinas were collected at P17 after treatment with SJW, hypericin, or vehicle for five days (P12-P17). Retinas were homogenized and sonicated in lysis buffer (Cell Signaling, Danvers, MA) with protease inhibitors (Sigma). Protein concentrations were measured using the BCA protein assay kit (Pierce, Rockford, IL), and the same amounts of retinal lysates were resolved by SDS–PAGE. Phospho-p42/44 ERK (Thr 202/Tyr 204) and ERK antibodies were purchased from Cell Signaling. β-actin antibody was purchased from Sigma. The membranes were immunoblotted with the indicated first antibodies at a 1:1000 dilution followed by the second antibodies conjugated with horseradish peroxidase at a 1:5000 dilution. Retinas were isolated from four mice per group. The same experiments were repeated three times.

### Statistical analysis

All values are presented as mean ± SEM. ANOVA was used to determine the differences among more than three groups. A level of p<0.05 was accepted as statistically significant.

## Results

### St. John’s Wort and hypericin inhibit retinal neovascularization in mice with ischemic retinopathy

**Figure 2:**
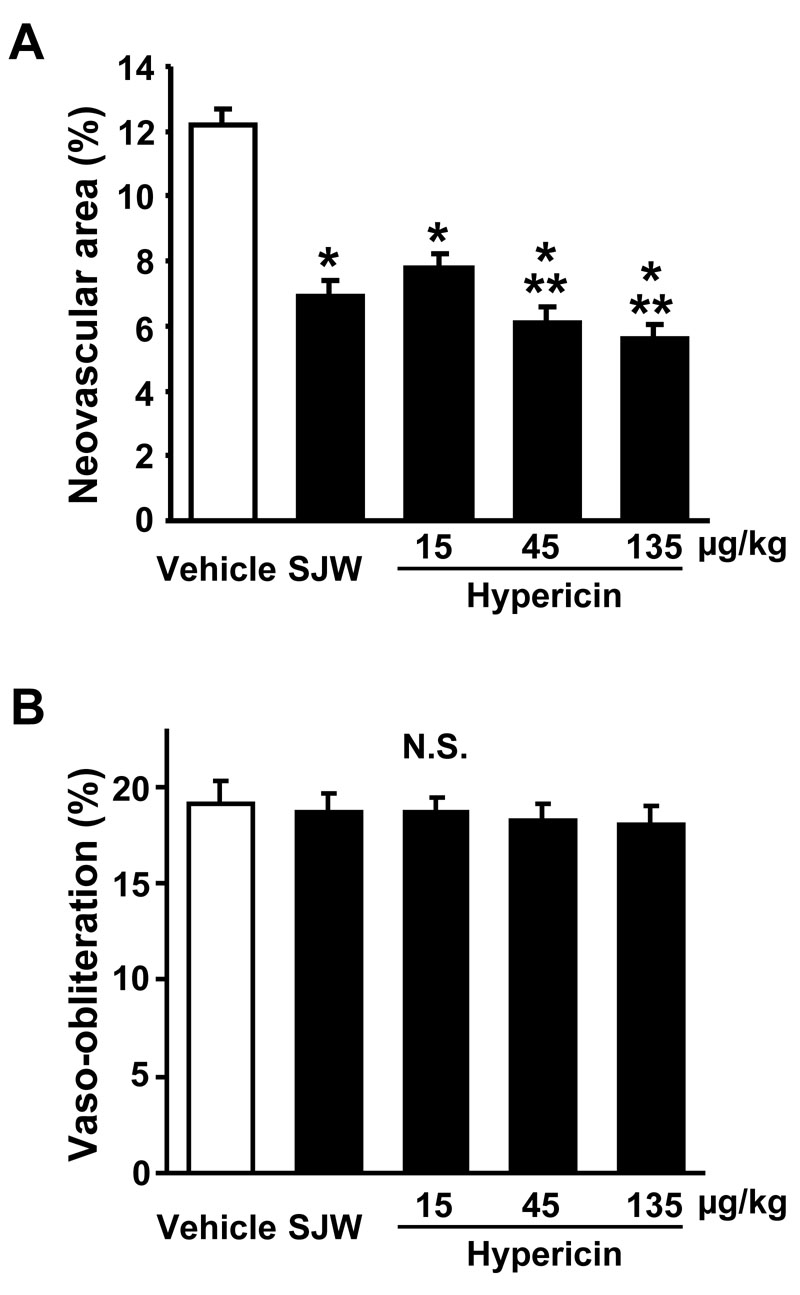
St. John’s Wort and hypericin inhibit retinal neovascularization. Quantification of neovascularization (A) and vasoobliteration (B) was performed after gavage treatment with either 15 mg/kg/day St. John’s Wort (SJW; n=18), 15 µg/kg/day hypericin (n=12), 45 µg/kg/day hypericin (n=18), 135 µg/kg/day hypericin (n=12), or vehicle (n=24). The percentages of retinal neovascularization and vasoobliteration were quantified by comparing the number of pixels in the affected areas with the total number of pixels in the retina. Data are presented as mean ± SEM. Asterisk (*) indicates p<0.01 versus vehicle treatment. Double asterisk (**) denotes p<0.05 versus hypericin (15 µg/kg/day) treatment. N.S. means the data was not statistically significant.

First, we investigated the impact of SJW (15 mg/kg/day) which approximately corresponds to the dose used as a supplement for humans, on ischemia-mediated retinal neovascularization in a mouse model of OIR. Representative photographs of isolectin B4-stained retinal vasculature in P17 mice are shown in Figure 1. Vehicle-treated mice exhibited a marked increase in multiple neovascular tufts, showing neovascularization throughout the retina (Figure 1A). In contrast, SJW-treated mice showed an apparent decrease in neovascularization compared with vehicle-treated mice (Figure 1B). Quantitative analyses revealed that oral SJW administration significantly reduced the area of neovascularization in retinas compared with vehicle (Figure 2A). The vasoobliteration area did not differ between the two groups (Figure 2B).

Because hypericin is one of major bioactive constituents of SJW, we also examined its effect on retinal neovascularization. Mice were treated with different doses of hypericin (15, 45, and 135 µg/kg/day). Hypericin treatment significantly inhibited the degree of retinal neovascularization in a dose-dependent fashion (Figure 1C, 2A). Treatment with hypericin at the dose of 15 µg/kg/day inhibited the retinal neovascularization to levels that were similar to that observed in SJW-treated mice (Figure 2A). The vasoobliteration area did not differ between hypericin-treated and vehicle-treated mice (Figure 2B).

### Retinal vascularization is not affected by treatment with St. John’s Wort and hypericin

**Figure 3:**
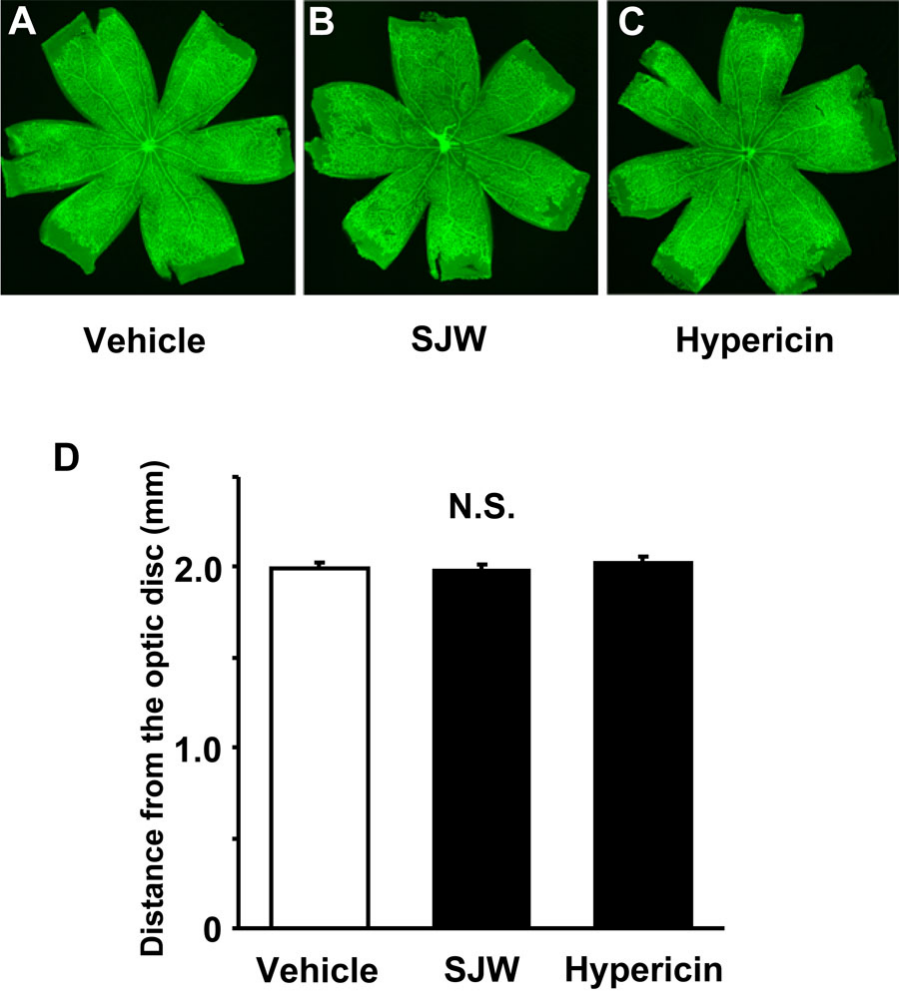
Effect of St. John’s Wort and hypericin on physiologic vascularization in the retina. A-C: Shown are representative images of mouse retinas immunostained with isolectin B4 after treatment with 15 mg/kg/day St. John’s Wort (SJW; n=18), 45 µg/kg/day hypericin (n=16), or vehicle (n=18) from P3 to P7. Magnification is X 5. D: Measurement of length of vasculature from the optic disc to the edge of the vascular front. Data are presented as mean ± SEM. N.S. means the data was not statistically significant.

Next, we tested whether SJW and hypericin influence physiologic retinal vessel growth as determined by staining with isolectin B4 at P7. Representative photographs of lectin-stained vessels in the retina of P7 mice are shown in Figure 3 A-C. Quantitative analysis was performed by measurement of the length from optic disc to the edge of retinal vessels. Both SJW and hypericin treatment had no effect on the length of vessels under these conditions (Figure 3D).

### St. John’s Wort and hypericin reduce extracellular signal-regulated kinase phosphorylation in retinas

**Figure 4:**
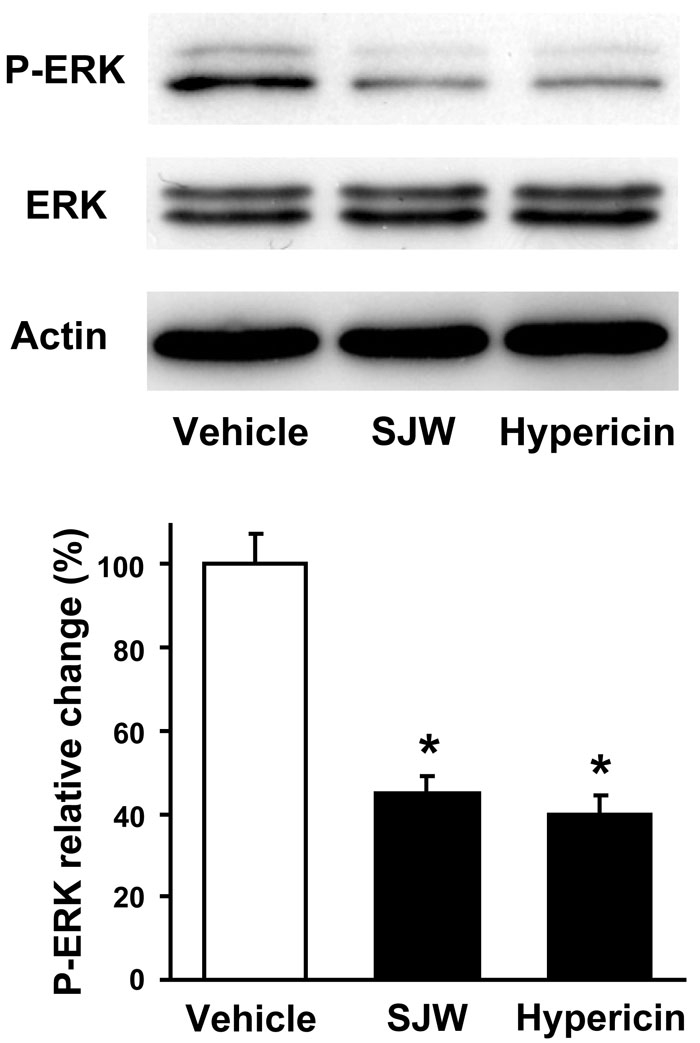
St. John’s Wort and hypericin inhibit extracellular signal-regulated kinase activation in retina. Retinas were harvested from eyes of mice following gavage administration of vehicle, 15 mg/kg/day St. John’s Wort (SJW) or 45 µg/kg/day hypericin from P12 through P17. Levels of phosphorylated ERK (P-ERK), total ERK, and actin were determined by western blot analysis. Representative blots from three experiments are shown. Phosphorylation levels of ERK were quantified by NIH image analysis. Immunoblots were normalized to total loaded protein. Data are presented as mean ± SEM. Asterisk (*) indicates p<0.01 versus vehicle treatment.

Because ERK plays important roles in the angiogenic responses, including retinal neovascularization [14,23], we used western blot analysis to assess the phosphorylation of ERK at Thr-202/Tyr-204 in retinas in each experimental group. SJW treatment markedly suppressed ERK phosphorylation in ischemic retina compared with vehicle (Figure 4). Similarly, ERK phosphorylation was diminished by treatment with hypericin (Figure 4). Both SJW and hypericin treatment had no effect on total ERK protein levels.

## Discussion

Our data provide in vivo evidence that SJW and its active ingredient, hypericin, inhibit the development of ischemic retinal neovascularization in a mouse model of ischemic retinopathy without affecting physiologic vascularization. Furthermore, the effects correlated with the ability of both SJW and hypericin to inhibit ERK phosphorylation under ischemic conditions. These data suggest that both SJW and hypericin could attenuate pathological retinal neovascularization through its ability to suppress ERK activation.

It has been shown that hypericin suppresses several angiogenic responses of bovine endothelial cells including endothelial growth, migration, and differentiation into vascular-like structure [24]. Hypericin also reduces proliferation and tube formation in choroidal endothelial cells [25]. Hypericin is reported to inhibit fibroblast growth factor -2-induced angiogenesis in a rat corneal assay in vivo [26]. Consistent with the antiangiogenic effects of hypericin under nonischemic conditions, the present study indicates that ischemia-induced retinal neovascularization is effectively diminished after treatment with hypericin. Collectively, these data suggest that hypericin can act as a general repressor of angiogenesis under pathological conditions.

In the present study, a therapeutic dose of SJW significantly attenuated retinal neovascularization in vivo. Hypericin also inhibited retinal neovascularization in a dose-dependent manner. Hypericin at the lowest dose was equally or less effective at inhibiting retinal neovascularization than SJW. Because this dose of hypericin was approximately proportional to that present in the dose of SJW, hypericin may partly account for the antiangiogenic actions of SJW. SJW also includes another active constituent, hyperforin, which is reported to mimic the antidepressive effects of SJW [17]. It has been reported that hyperforin inhibits vessel growth in vivo determined by chorioallantoic membrane assay, and that it also suppresses angiogenic responses including proliferation and tube-like structure formation in cultured endothelial cells [27]. In addition, SJW contains several flavonoids, such as rutin and quercetin. These flavonoids may also inhibit angiogenesis in vivo and in vitro [28,29]. Thus, SJW has several active components showing antiangiogenic properties.

VEGF is critical for the determination of neovascularization in ischemic ocular diseases, including diabetic retinopathy [2,5,15], and inhibition of VEGF signaling results in reduction of retinal angiogenesis [6,14,15,30,31]. VEGF stimulation leads to ERK activation in endothelial cells [7-9]. In this regard, hypericin has been shown to suppress ERK activation in both cancer and endothelial cell lines [18,26]. In line with these observations, our data show that hypericin suppressed ERK phosphorylation in a mouse model of OIR. Therefore, the inhibitory actions of hypericin on neovascularization may be mediated partly by its ability to suppress VEGF-stimulated signaling.

In summary, we show that both hypericin and SJW protect against pathological retinal neovascularization in mice with ischemic retinopathy. The beneficial effects of SJW and hypericin are associated with reduced ERK activation in retina. Thus, these agents could be potentially useful for treatment of ischemic ocular diseases.

## References

[1] Carmeliet P (2003). Angiogenesis in health and disease.. Nat Med.

[2] Pierce EA, Avery RL, Foley ED, Aiello LP, Smith LE (1995). Vascular endothelial growth factor/vascular permeability factor expression in a mouse model of retinal neovascularization.. Proc Natl Acad Sci USA.

[3] Smith LE (2003). Pathogenesis of retinopathy of prematurity.. Semin Neonatol.

[4] Kvanta A (2006). Ocular angiogenesis: the role of growth factors.. Acta Ophthalmol Scand.

[5] Ishida S, Usui T, Yamashiro K, Kaji Y, Amano S, Ogura Y, Hida T, Oguchi Y, Ambati J, Miller JW, Gragoudas ES, Ng YS, D'Amore PA, Shima DT, Adamis AP (2003). VEGF164-mediated inflammation is required for pathological, but not physiological, ischemia-induced retinal neovascularization.. J Exp Med.

[6] Ozaki H, Seo MS, Ozaki K, Yamada H, Yamada E, Okamoto N, Hofmann F, Wood JM, Campochiaro PA (2000). Blockade of vascular endothelial cell growth factor receptor signaling is sufficient to completely prevent retinal neovascularization.. Am J Pathol.

[7] Takahashi T, Yamaguchi S, Chida K, Shibuya M (2001). A single autophosphorylation site on KDR/Flk-1 is essential for VEGF-A-dependent activation of PLC-gamma and DNA synthesis in vascular endothelial cells.. EMBO J.

[8] Takahashi T, Ueno H, Shibuya M (1999). VEGF activates protein kinase C-dependent, but Ras-independent Raf-MEK-MAP kinase pathway for DNA synthesis in primary endothelial cells.. Oncogene.

[9] Wong C, Jin ZG (2005). Protein kinase C-dependent protein kinase D activation modulates ERK signal pathway and endothelial cell proliferation by vascular endothelial growth factor.. J Biol Chem.

[10] Shiojima I, Walsh K (2002). Role of Akt signaling in vascular homeostasis and angiogenesis.. Circ Res.

[11] Xia P, Aiello LP, Ishii H, Jiang ZY, Park DJ, Robinson GS, Takagi H, Newsome WP, Jirousek MR, King GL (1996). Characterization of vascular endothelial growth factor's effect on the activation of protein kinase C, its isoforms, and endothelial cell growth.. J Clin Invest.

[12] Yoshiji H, Kuriyama S, Ways DK, Yoshii J, Miyamoto Y, Kawata M, Ikenaka Y, Tsujinoue H, Nakatani T, Shibuya M, Fukui H (1999). Protein kinase C lies on the signaling pathway for vascular endothelial growth factor-mediated tumor development and angiogenesis.. Cancer Res.

[13] Kroll J, Waltenberger J (1997). The vascular endothelial growth factor receptor KDR activates multiple signal transduction pathways in porcine aortic endothelial cells.. J Biol Chem.

[14] Bullard LE, Qi X, Penn JS (2003). Role for extracellular signal-responsive kinase-1 and −2 in retinal angiogenesis.. Invest Ophthalmol Vis Sci.

[15] Aiello LP, Pierce EA, Foley ED, Takagi H, Chen H, Riddle L, Ferrara N, King GL, Smith LE (1995). Suppression of retinal neovascularization in vivo by inhibition of vascular endothelial growth factor (VEGF) using soluble VEGF-receptor chimeric proteins.. Proc Natl Acad Sci USA.

[16] Di Carlo G, Borrelli F, Ernst E, Izzo AA (2001). St John's wort: Prozac from the plant kingdom.. Trends Pharmacol Sci.

[17] Barnes J, Anderson LA, Phillipson JD (2001). St John's wort (Hypericum perforatum L.): a review of its chemistry, pharmacology and clinical properties.. J Pharm Pharmacol.

[18] Assefa Z, Vantieghem A, Declercq W, Vandenabeele P, Vandenheede JR, Merlevede W, de Witte P, Agostinis P (1999). The activation of the c-Jun N-terminal kinase and p38 mitogen-activated protein kinase signaling pathways protects HeLa cells from apoptosis following photodynamic therapy with hypericin.. J Biol Chem.

[19] Takahashi I, Nakanishi S, Kobayashi E, Nakano H, Suzuki K, Tamaoki T (1989). Hypericin and pseudohypericin specifically inhibit protein kinase C: possible relation to their antiretroviral activity.. Biochem Biophys Res Commun.

[20] Connor KM, SanGiovanni JP, Lofqvist C, Aderman CM, Chen J, Higuchi A, Hong S, Pravda EA, Majchrzak S, Carper D, Hellstrom A, Kang JX, Chew EY, Salem N, Serhan CN, Smith LE (1994). Oxygen-induced retinopathy in the mouse.. Invest Ophthalmol Vis Sci.

[21] Butterweck V, Schmidt MSt (2007). John's wort: role of active compounds for its mechanism of action and efficacy.. Wien Med Wochenschr.

[22] Connor KM, SanGiovanni JP, Lofqvist C, Aderman CM, Chen J, Higuchi A, Hong S, Pravda EA, Majchrzak S, Carper D, Hellstrom A, Kang JX, Chew EY, Salem N, Serhan CN, Smith LE (2007). Increased dietary intake of omega-3-polyunsaturated fatty acids reduces pathological retinal angiogenesis.. Nat Med.

[23] Reddy KB, Nabha SM, Atanaskova N (2003). Role of MAP kinase in tumor progression and invasion.. Cancer Metastasis Rev.

[24] Martinez-Poveda B, Quesada AR, Medina MA (2005). Hypericin in the dark inhibits key steps of angiogenesis in vitro.. Eur J Pharmacol.

[25] Kimura H, Harris MS, Sakamoto T, Gopalakrishna R, Gundimeda U, Cui JZ, Spee C, Hinton DR, Ryan SJ (1997). Hypericin inhibits choroidal endothelial cell proliferation and cord formation in vitro.. Curr Eye Res.

[26] Lavie G, Mandel M, Hazan S, Barliya T, Blank M, Grunbaum A, Meruelo D, Solomon A (2005). Anti-angiogenic activities of hypericin in vivo: potential for ophthalmologic applications.. Angiogenesis.

[27] Martinez-Poveda B, Quesada AR, Medina MA (2005). Hyperforin, a bio-active compound of St. John's Wort, is a new inhibitor of angiogenesis targeting several key steps of the process.. Int J Cancer.

[28] Schindler R, Mentlein R (2006). Flavonoids and vitamin E reduce the release of the angiogenic peptide vascular endothelial growth factor from human tumor cells.. J Nutr.

[29] Tan WF, Lin LP, Li MH, Zhang YX, Tong YG, Xiao D, Ding J (2003). Quercetin, a dietary-derived flavonoid, possesses antiangiogenic potential.. Eur J Pharmacol.

[30] Suzuma K, Takahara N, Suzuma I, Isshiki K, Ueki K, Leitges M, Aiello LP, King GL (2002). Characterization of protein kinase C beta isoform's action on retinoblastoma protein phosphorylation, vascular endothelial growth factor-induced endothelial cell proliferation, and retinal neovascularization.. Proc Natl Acad Sci USA.

[31] Ojima T, Takagi H, Suzuma K, Oh H, Suzuma I, Ohashi H, Watanabe D, Suganami E, Murakami T, Kurimoto M, Honda Y, Yoshimura N (2006). EphrinA1 inhibits vascular endothelial growth factor-induced intracellular signaling and suppresses retinal neovascularization and blood-retinal barrier breakdown.. Am J Pathol.

